# Prediction of COVID-19 diagnosis based on openEHR artefacts

**DOI:** 10.1038/s41598-022-15968-z

**Published:** 2022-07-22

**Authors:** Daniela Oliveira, Diana Ferreira, Nuno Abreu, Pedro Leuschner, António Abelha, José Machado

**Affiliations:** 1 Algoritmi Research Center, University of Minho, Campus of Gualtar, Braga, 4710 Portugal; 2 Centro Hospitalar Universitário do Porto, Porto, 4099 Portugal

**Keywords:** Viral infection, Preventive medicine, Epidemiology

## Abstract

Nowadays, we are facing the worldwide pandemic caused by COVID-19. The complexity and momentum of monitoring patients infected with this virus calls for the usage of agile and scalable data structure methodologies. OpenEHR is a healthcare standard that is attracting a lot of attention in recent years due to its comprehensive and robust architecture. The importance of an open, standardized and adaptable approach to clinical data lies in extracting value to generate useful knowledge that really can help healthcare professionals make an assertive decision. This importance is even more accentuated when facing a pandemic context. Thus, in this study, a system for tracking symptoms and health conditions of suspected or confirmed SARS-CoV-2 patients from a Portuguese hospital was developed using openEHR. All data on the evolutionary status of patients in home care as well as the results of their COVID-19 test were used to train different ML algorithms, with the aim of developing a predictive model capable of identifying COVID-19 infections according to the severity of symptoms identified by patients. The CRISP-DM methodology was used to conduct this research. The results obtained were promising, with the best model achieving an accuracy of 96.25%, a precision of 99.91%, a sensitivity of 92.58%, a specificity of 99.92%, and an AUC of 0.963, using the Decision Tree algorithm and the Split Validation method. Hence, in the future, after further testing, the predictive model could be implemented in clinical decision support systems.

## Introduction

Today’s Health Information Systems (HIS) include numerous types of software, resulting in a wide range of versions and technologies employed, even within the same organisation. Because of the lack of national and institutional guidelines, different parts of a given HIS represent the same information in different ways, posing a significant barrier to semantic interoperability. Information Models (IMs) capable of lowering the barriers that have accumulated over time to achieve HIS interoperability have become highly significant. Existing global standards for the development of consistent and interoperable IMs are becoming increasingly important in this endeavour. Such standards can cover demographic, clinical, and administrative modules, as well as information access control.

All of these issues jeopardise the ability of Information Technology (IT) to help improve daily clinical practice and valuable knowledge production, as well as limit the ability to deploy reliable Clinical Decision Support Systems (CDSSs)^1,2^. Clinical data not only enables decisions for continuity of care, but also serves several secondary uses, such as medical and academic research, business intelligence indicators, and the discovery of new knowledge using Artificial Intelligence (AI) and Machine Learning (ML) algorithms. Although secondary, these applications are considered critical for continuous improvement in the delivery of quality healthcare. Hence, it is necessary to provide support for new data sources, including wearables, patient reported data, and external systems.

Therefore, it is of uttermost importance that distinct data representations are transformed and integrated according to a common data model^3–5^.

COVID-19 (Coronavirus disease of 2019) is the name given by the World Health Organisation (WHO) to the infectious disease caused by SARS-CoV2, the most recently discovered coronavirus, which is infecting large numbers of people worldwide and for which no country was prepared. As a result, many countries’ healthcare systems became overburdened during the COVID-19 pandemic. This was due to a variety of factors, including lack of human and material capital while the demand for healthcare was increasing. Furthermore, the difficulties encountered in exchanging data in regular day-to-day work were exacerbated by the pandemic’s pressure, putting even more strain on healthcare professionals. Medical data is a valuable resource in these emergency situations, not only for clinical decisions but also for health political governance, since it provides up-to-date and real-time information on the pandemic’s progression.

**Figure 1:**
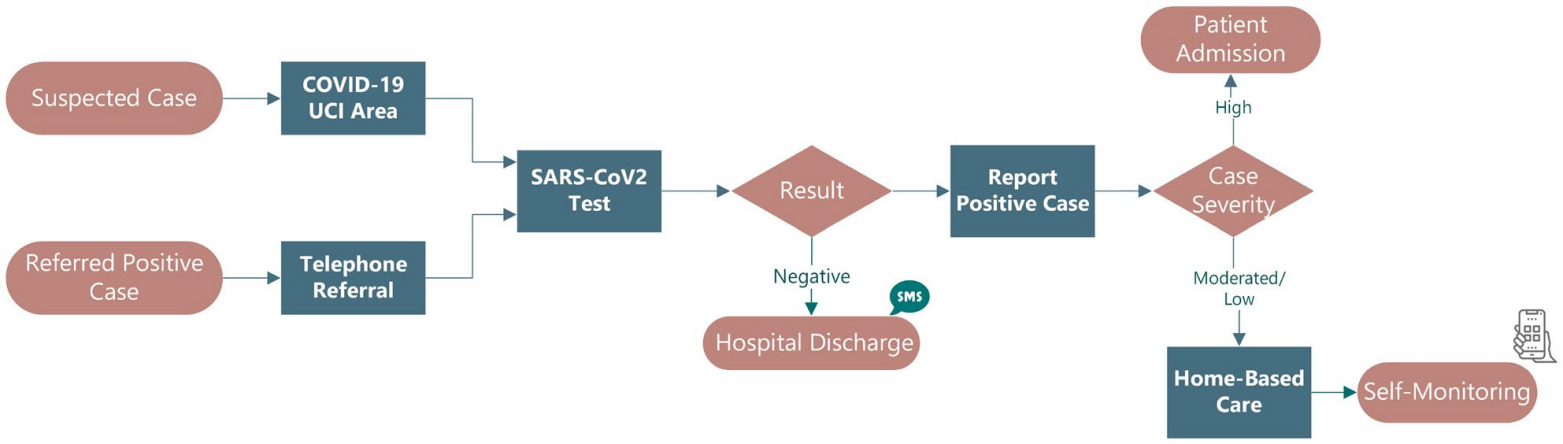
Flow of the referenced or suspected COVID-19 patient.

An ongoing pilot project focused on the openEHR (open Eletronic Health Records) specification was used by Centro Hospitalar Universitário do Porto (CHUP) institution to refine the COVID-19 patient’s treatment workflow, which is depicted in Fig. 1. As a result, a hybrid solution that interacted with existing Legacy Systems (LS) was created to ensure that users could communicate quickly and effectively with the least amount of effort. In addition, a web application has been developed to keep track of patients receiving home care. The data entry of this application was ensured through a form based on a template modelled in openEHR’s clinical methodology. This tool was developed for symptomatic patients to determine whether they have COVID-19. Each patient was free to report their symptoms and health status as many times as they wanted, either before or after learning the results of their SARS-CoV-2 test within a 14 days time window^6,7^.

In this context, the main motivation for this article focuses on exploring and exploiting the collected data in order to develop and train ML models capable of predicting patients infected with COVID-19, thereby assisting healthcare institutions to deal with the new cases of infection. The Cross Industry Standard Process for Data Mining (CRISP-DM) methodology, used for the development of Data Mining (DM) processes, was adopted to ensure that the models created are valid and replicable.

The following document is structured in five chapters. The first chapter makes a contextualisation of the topic addressed in this work and presents its main motivations and objectives. In the next chapter, all theoretical and scientific concepts of interest for this document are presented and documented. Following, the paper contains the methodology chapter, which includes the CRISP-DM methodology and all steps performed. Next, the results are presented and its discussion is made. Finally, the last chapter summarises and presents the main conclusions and contributions obtained through the development of this platform, as well as the proposals for future work.

### Interoperable healthcare systems and the use of the openEHR standard

Interoperability is defined as the ability of two or more systems to communicate with one another without requiring additional effort from the user, sharing critical data and initiating actions on one another^8^. Any healthcare environment is made up of a variety of different types of care that are provided by various departments and facilities. These processes are notoriously complex and paper-based, which HIS and technological advancements seek to address. However, as medical and information technology become more sophisticated, the risk of medical errors increases^9^. The range, format, value set, occurrence, and cardinality of data must be ensured in all exchanges and modifications made over time.

The openEHR standard has already demonstrated its worth and adaptability in a variety of dynamically changing situations. It is based on a two-level knowledge modelling approach in which the Clinical Information Model (CIM) is built independently of the Reference Model (RM), separating clinical and technological domains and allowing for more autonomous development in each of these domains^10,11^.

The CIM promotes information consistency in the clinical domain, providing structural interoperability by using archetype units as basic components. These, in turn, are used to model increasingly complex structures, which are known as templates. These templates are easily adaptable to a given clinical environment by reusing existing archetypes and creating new ones for the representation of concepts that were not previously modelled.

Standardisation and the building block approach are especially effective in hospital settings, where the involvement of experts from each of the different areas is required for data visualisations and data entry forms to be consistent with the specific setting^1,2,12–15^. On one hand, the specification of concepts through the use of terminologies and clinical guidelines ensures semantic compatibility. On the other hand, RM incorporates a set of classes that describe the generic structure of a patient’s Eletronic Health Record (EHR), context and audit details, all versioning standards, and access to archetypes data through *locatable* class and datatype declarations to ensure syntactic interoperability^16,17^.

### Machine learning to predict diseases

The huge volumes of data generated in a hospital setting on a daily basis demand mechanisms to classify or cluster them^18^. As a result, ML has become the most widely used sub-field of AI, with techniques including reinforcement learning and deep learning. Several everyday applications use AI recommendation systems, which apply a variety of ML methods to generate classifiers, clusters, and rules that can organise all data based on its attributes. Efforts are being made to ensure that this trend continues in healthcare.

In literature, different healthcare and nutrition projects have used ML systems with a variety of algorithms. DM studies also use ML approaches to extract knowledge. In^19^, the authors have made a survey work about the diseases diagnosed by ML techniques, such as diabetes, heart failure, hepatitis, etc. The authors have noted that Naïve Bayes (NB) and Support Vector Machines (SVM) algorithms can be successfully implemented to predict diseases, offering the best accuracy compared to tree algorithms. Another review paper was written in^20^ in the context of the human microbiome. Several works developed using ML techniques for forecasting diagnoses such as Crohn’s and colorectal diseases, bacterial vaginosis, colorectal cancer, obesity, and allergies, among others, were subjected to a systematic review. In terms of user applicability, an interactive web application for diabetes prediction was developed in^21^ using the Pima Indian benchmark dataset to train an Artificial Neural Network (ANN). In order to predict a diagnosis, the application records some relevant information about the users as an input to an inference system, such as glucose and blood pressure values, body mass index, age, etc.

The work published in *Applications of Data Mining Techniques in Healthcare and Prediction of Heart Attacks* has implemented another DM study in a healthcare environment. Their main goal was to apply DM techniques for the prediction of heart attacks using medical profiles such as age, sex, blood pressure, and sugar levels. By using ODANB classifiers and NCC2, they were successfully able to create models that predict the risk of heart attacks^22^.

Additionally, the authors of *ML in Nutritional Follow-up Research* show how a DM study was developed using a nutritional dataset and ML algorithms. The main purpose of this case study is to create a predictive model for the eventual necessity of a patient to be followed by a nutrition specialist. The CRISP-DM method was combined with data from CHUP institution. Furthermore, five ML models were tested, including Decision Trees (DT), SVM, Bayesian Networks (BN), Decision Rules (DR), and Nearest Neighbours (NN). The researchers developed multiple models, finding the key features of a patient that predicted the need for follow-up by a nutrition specialist, thereby assisting physicians in making the optimal choices^23^.

## Related work

Several national healthcare systems and hospitals have adopted the openEHR standard methodology. The Ministry of Health in the Republic of Slovenia is currently implementing openEHR standards in a large scale. he developed solution is an HIS capable of transforming data into the openEHR format and supporting healthcare systems from LSs. As of the publication of this document, more than 85% of Slovenia’s national heath data is saved on the developed platform^24^. Additionally, Wales’s National Health Service (NHS) has been conducting a technical evaluation of openEHR’s ability as a repository for structured clinical data, aiming to roll it out to support national projects and provide shared medication records for NHS Wales^25^.

Some research has been carried to determine the adaptability of openEHR to specific medical domains, such as obstetrics department. A study was conducted to determine the most adaptable strategy to respond to the new needs of Obscare users, a 15-year-old software. The authors proposed openEHR as a viable methodology capable of representing an obstetric-specific EHR as well as clinical principles. Their analysis reveals that openEHR’s Clinical Knowledge Manager (CKM) repository requires additional work to completely fulfil the demands of obstetrics. There are yet obstetric archetypes to model, and modifications to those that already exist may be necessary^26^.

Several studies advocate the use of architectures based on the dual model approach as an alternative to traditional information models, splitting the clinical semantics associated to the information and knowledge into two distinct levels. The openEHR Modeling Methodology (openEHR-MM), created by Afef S. Ellouzea, Sandra H. Tlilia, and Rafik Bouazizb, is a novel approach for building interfaces based on openEHR archetypes.Their artefact was tested on the scenario of children with cerebral palsy and, the authors mention, as a future work, the use of ontologies to map the Unified Modeling Language (UML) diagram of RM^27^.

In terms of Internet of Things (IoT) devices, the authors of *Open IoT architecture for continuous patient monitoring in emergency wards* have proposed an open architecture to track the patients’ physiological parameters, using open protocols from wearable sensors to the monitoring system. In the IoT device aspect, the authors rely on the oneM2M (one Machine to Machine) technical standard for interoperability regarding architecture, Application Programming Interface (API) specifications, and security for M2M/IoT technologies, while employing openEHR for data semantics, storage, and making health data available to professionals in their EHR^28^.

The advanced mindset in the Netherlands shows the importance of a patient-centered approach healthcare. At the level of IT infrastructure flexibility, Tarenskeen, Debbie and van de Wetering, Rogier and Bakker, René and Brinkkemper, Sjaak argue that Conceptual Independence (CI) contributes to flexible data models that are independent of the application side. Their study was carried out using mixed-methods research in ten healthcare organisations, five of which had implemented openEHR. When it comes to demonstrating that systems based on openEHR have a greater capacity for change and remodelling, all of the studies came to the same conclusion. In addition, these organisations have demonstrated a positive impact on functionality reuse and modularity^29^. The openEHR methodology cannot be used exclusively to build a healthcare system that stimulates a collaborative work method between clinical and IT experts in order to create a standardised and reliable system free of data loss.

To ensure the integrity of the data from the LS, the research entitled by *A Migration Methodology from Legacy to New Electronic Health Record Based on OpenEHR* suggests an interoperable approach to convert Structured Query Language (SQL) architecture to a NoSQL (Not only SQL) scheme, maintaining the integrity of clinical data^30^.

In order to create intelligent systems, in *Automatic Conversion of Electronic Medical Record Text for OpenEHR Based on Semantic Analysis*, the authors proposed a Wide and Deep Recurrent Neural Network (WDRNN) algorithm to convert automatically free text into structured electronic records, based on the ML classification approach. Besides, the authors also presented a Conditional Random Fields as Recurrent Neural Networks (CRF-RNN) Label Model (CRLM) to improve the accuracy of entities and clinical concepts^31^.

## Methods

SARS-CoV-2, the newest coronavirus, caused an unexpected global epidemic in late 2019 and early 2020. As the pandemic spread, each country’s healthcare system had to adapt fast to new cases. As a contingency plan, COVID-19 screening questions based on clinical symptoms were included. This study proposes an openEHR-based system for tracking symptoms and health conditions of suspected or confirmed SARS-CoV-2 infected patients. This system was implemented in the CHUP institution, and patients could use it to track their clinical status at home either before or after learning their SARS-CoV-2 test result. The main focus of the present study is to extract useful patterns and knowledge from the data of inquired patients in the hopes of developing a predictive model capable of distinguishing between healthy people and people with COVID-19, thus assisting healthcare professionals in the early detection of infected patients, allowing them to isolate as soon as possible and consequently decreasing the spread of the virus. To perform a more detailed analysis and extract new knowledge about Portugal’s epidemiological situation from the data generated by the novel system, the CRISP-DM methodology was determined to be appropriate for its processing and analysis.

### Ethics

Between March 2020 and January 2021, all patients who submitted data from the dataset under study gave their consent for their data to be processed in accordance with the provisions of article 9, paragraph 2, c) of the Portuguese General Data Protection Regulation (GDPR) and article 29 of law 58/2019 (Portuguese national law implementing the GDPR), as well as their consent for the use of the *CHUPCovid* application, article 9, paragraph 2, a) of the GDPR of the Portuguese legislation^32^.

The authors were responsible for the data acquisition and processing software, according to CHUP hospital requirements. The study was reviewed by the interdisciplinary Hospital’s Information Technology Committee, in Portuguese *Comissão de Informática*, which checked data and procedural compliance with current ethical and legal guidelines. In this research, data anonymisation was also used to ensure security and privacy of the personal data of the patients involved. In the data collection stage of this study, the information that could compromise the patient’s identification was removed or transformed accordingly. This method produced completely anonymised data that cannot be linked to any specific individual. Furthermore, the authors have obtained permission from the CHUP institution’s president to use the dataset and publish the study’s findings. A physician and a nurse, both authors of the current paper, also guaranteed that the hospital’s ethical and data protection rules were followed.

### CRISP-DM

**Figure 2:**
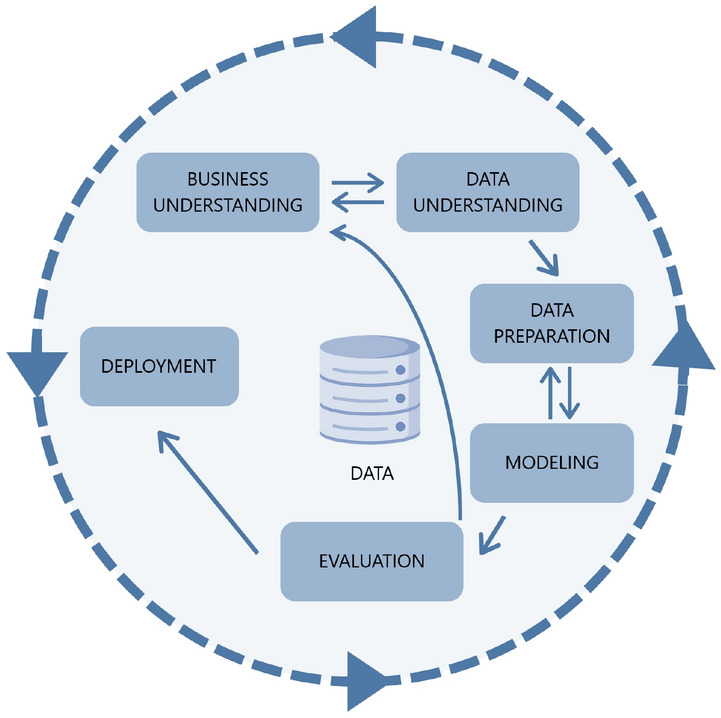
Stages of the CRISP-DM methodology. Adapted from^34^.

CRISP-DM is a popular framework used for developing DM processes worldwide. This methodology was financed by the European Community and allows for project replication as well as project planning and management^33^. CRISP-DM is a cyclical process comprised of six stages, as shown in Fig. 2: Business Understanding, Data Understanding, Data Preparation, Modelling, Evaluation, and Deployment^34^. The next subsections contain a detailed description of each of these phases, with the exception of the last phase, the deployment of the model.

#### Business understanding

COVID-19 symptoms include fever, headache, respiratory symptoms such cough and dyspnea, and loss of taste or smell. Because it is a new disease with variable manifestations, diagnosing it is challenging as most of the symptoms are mild, common daily conditions for some people, such as headaches, or symptoms of common diseases, such as the flu. For this reason, COVID-19 is solely diagnosed through laboratory tests. Thus, a model that can detect COVID-19 based on patient’s clinical symptoms could greatly improve the health system’s ability to respond quickly and efficiently to new cases. The purpose of this study is to investigate which factors influence COVID-19 diagnosis and to develop a predictive model for early disease detection using patients’ clinical symptomatology data. To build models that can extract relevant information from patient data, this study will apply different ML algorithms.

#### Data understanding

In order to fully understand the data and discover relationships between attributes, it was essential to go through this stage. The dataset used in this study contains a variety of information extracted from an openEHR-based system for tracking symptoms and health conditions of suspected or confirmed SARS-CoV-2 infected patients being treated at the CHUP. The data in the anonymised dataset under study only refers to patient submissions made between March 2020 and January 2021, for a total of 13,434 instances and 14 attributes. Each instance corresponds to an enquired patient and contains his/her medical data. The dataset under study is composed of 4 integer attributes, 9 polynomial attributes, and 1 binomial attribute that corresponds to the COVID-19 test result. The attributes are described in the Table 1. The values of each polynomial and binomial attribute are presented in the footnotes of the table, with the exception of the *Medication_last_24h* attribute, which contains several different medication types available, such as “Paracetamol”, “Omeprazol”, “Ibuprofeno”, among others.

**Table 1:** Description of the attributes of the dataset under study

Attribute	Description	Type
Patient_id	Patient’s Identifier	Integer
Age	Patient’s age	Integer
Gender	Patient’s gender^1^	Integer
Temperature	Patient’s body temperature^2^	Integer
Headache	Patient’s headache evaluation^2^	Polynomial
Muscle_pain	Patient’s muscle pain evaluation^2^	Polynomial
Cough	Patient’s cough evaluation^2^	Polynomial
Diarrhea	Patient’s diarrhea evaluation^2^	Polynomial
Thoracalgia	Patient’s thoracalgia evaluation^2^	Polynomial
Shortness_of_breath	Patient’s shortness of breath evaluation^2^	Polynomial
Shortness_of_smell_taste	Patient’s shortness of smell and taste evaluation^2^	Polynomial
Medication_last_24h	Medications taken in the previous 24 hours	Polynomial
Global_evaluation	Patient’s health status^3^	Polynomial
Result	COVID-19 test result^4^	Binomial

^1^{Female, Male} ^2^{No,I have now, Keeps, Improved, Worsened}  ^3^{I feel better, I feel worse, I feel the same} ^4^{Negative, Positive}.

The following figures depict the patient’s data distribution in terms of age, gender, and temperature.

**Figure 3:**
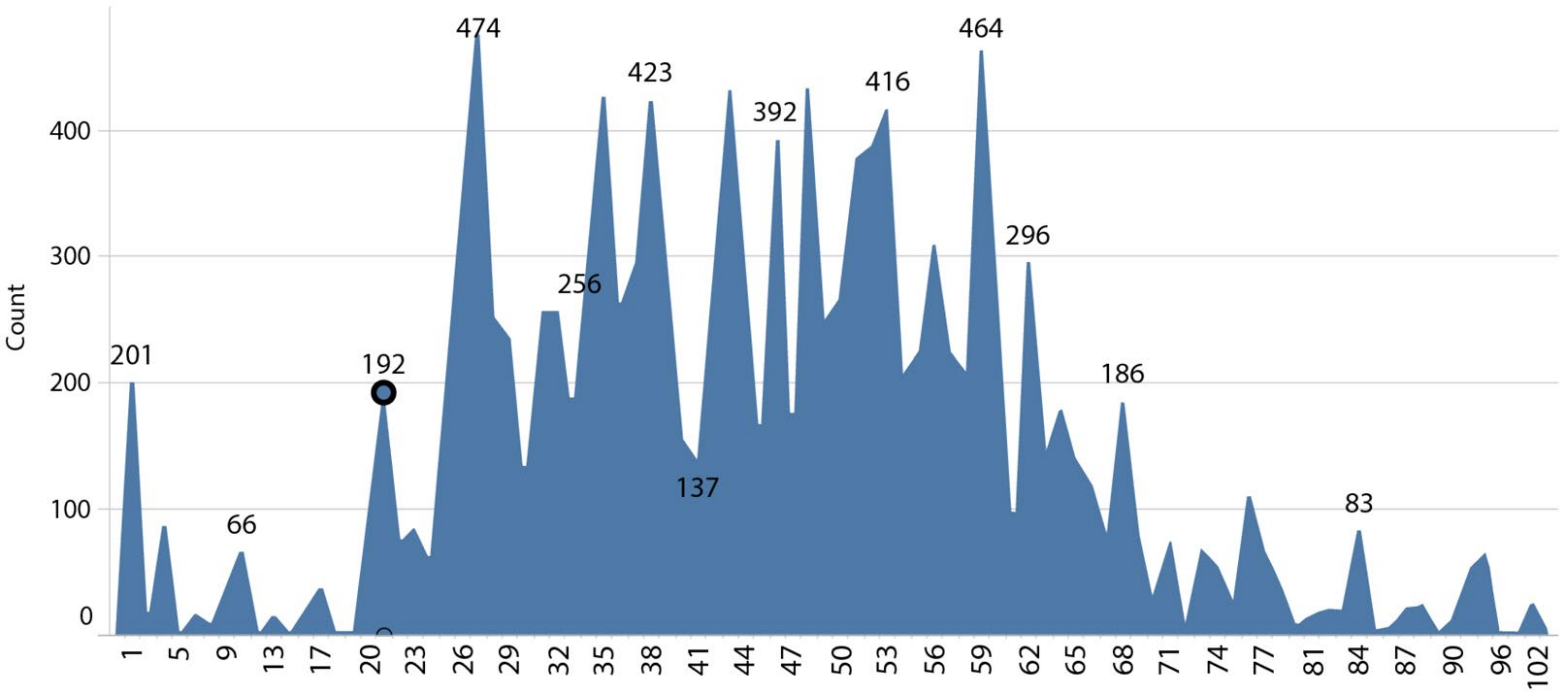
Distribution of patients per age.

Figure 3 represents the age distribution from newborns to the elderly, with the age range between 25 and 60 years containing the greatest number of records. The fact that the COVID-19 screening questionnaires were available online, suggests that the higher concentration of information in this age group may be due to its greater familiarity with modern technology.

**Figure 4:**
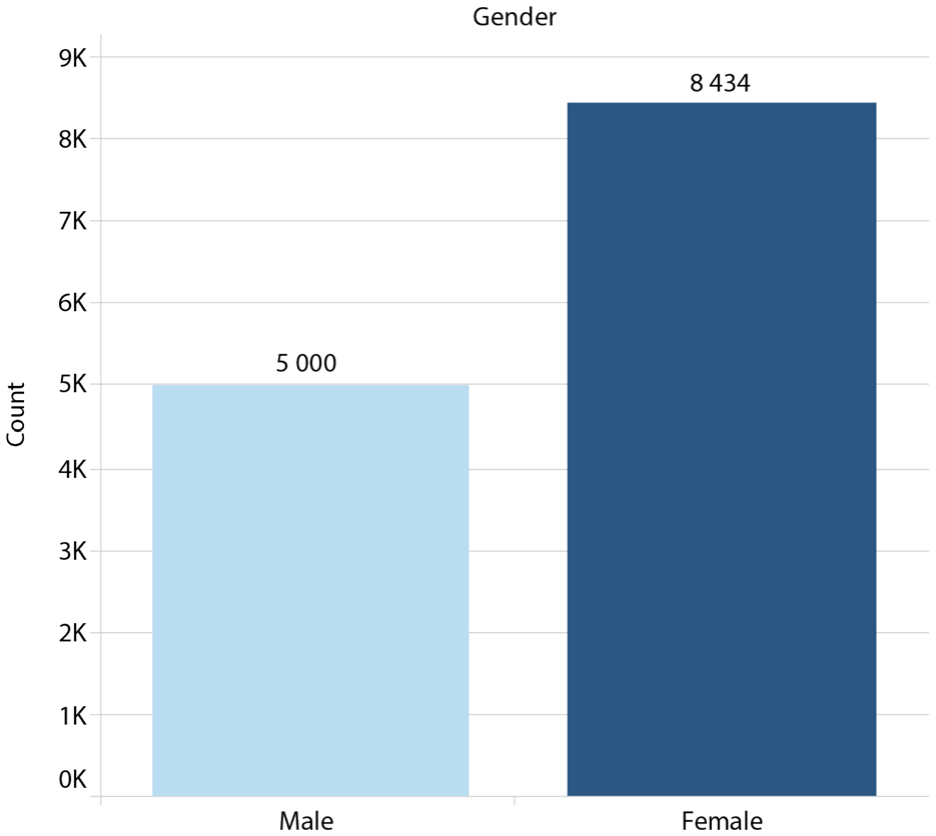
Distribution of patients per gender.

In terms of patient’s gender, Fig. 4 shows that the female gender was more prevalent than the male gender in the research dataset.

**Figure 5:**
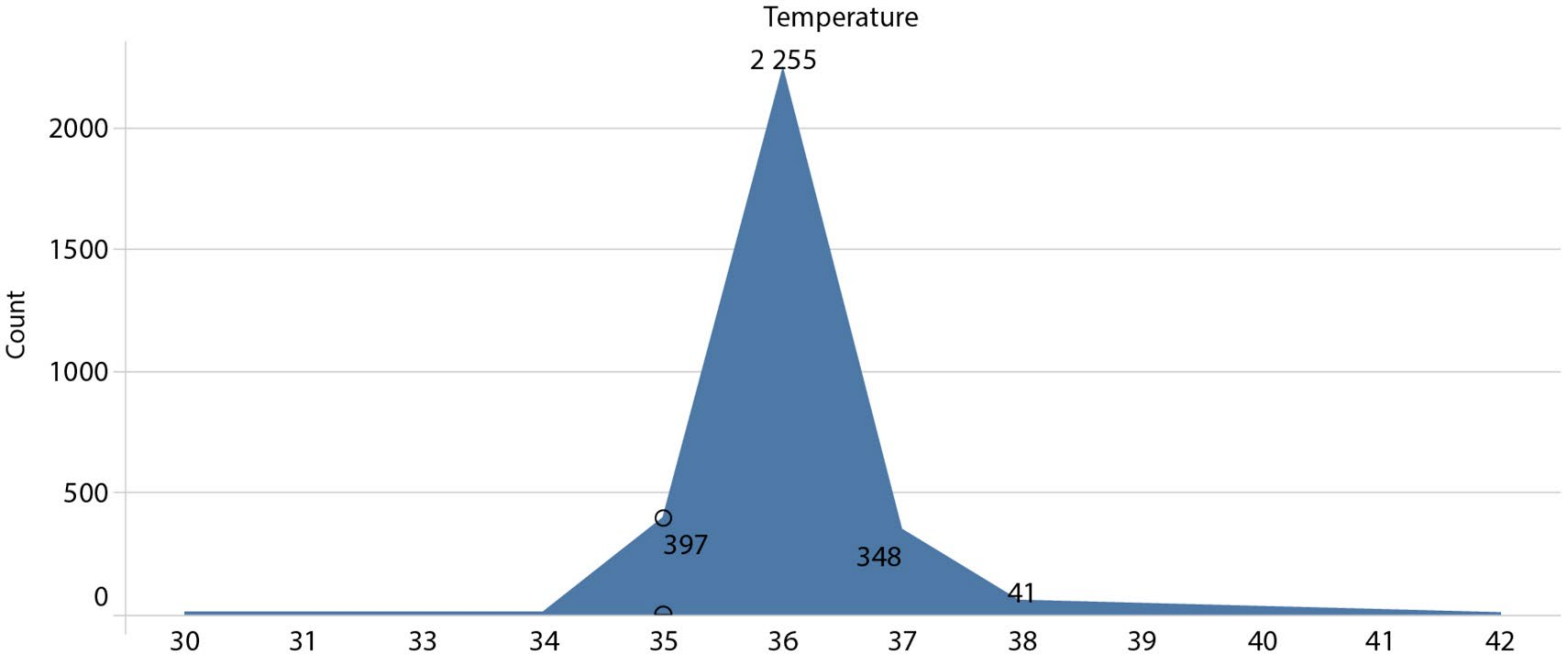
Distribution of patients per body temperature.

One of the most important established metrics is the patient’s body temperature, which was calculated by the patient himself/herself. Figure 5 describes the temperature distribution of the patients, ranging from 35 to 42 degrees Celsius.

Figure 5 analysis reveals that the majority of patients have a temperature of 36 degrees Celsius, and by plotting a perpendicular axis at that point, a nearly symmetrical distribution of temperatures to either side can be seen. Unexpectedly, most patients have a normal body temperature, concentrating between 35 and 37 degrees Celsius. This is surprising given that the majority of the individuals considered in this study are COVID-19 positive cases, as it will be seen below.

**Figure 6:**
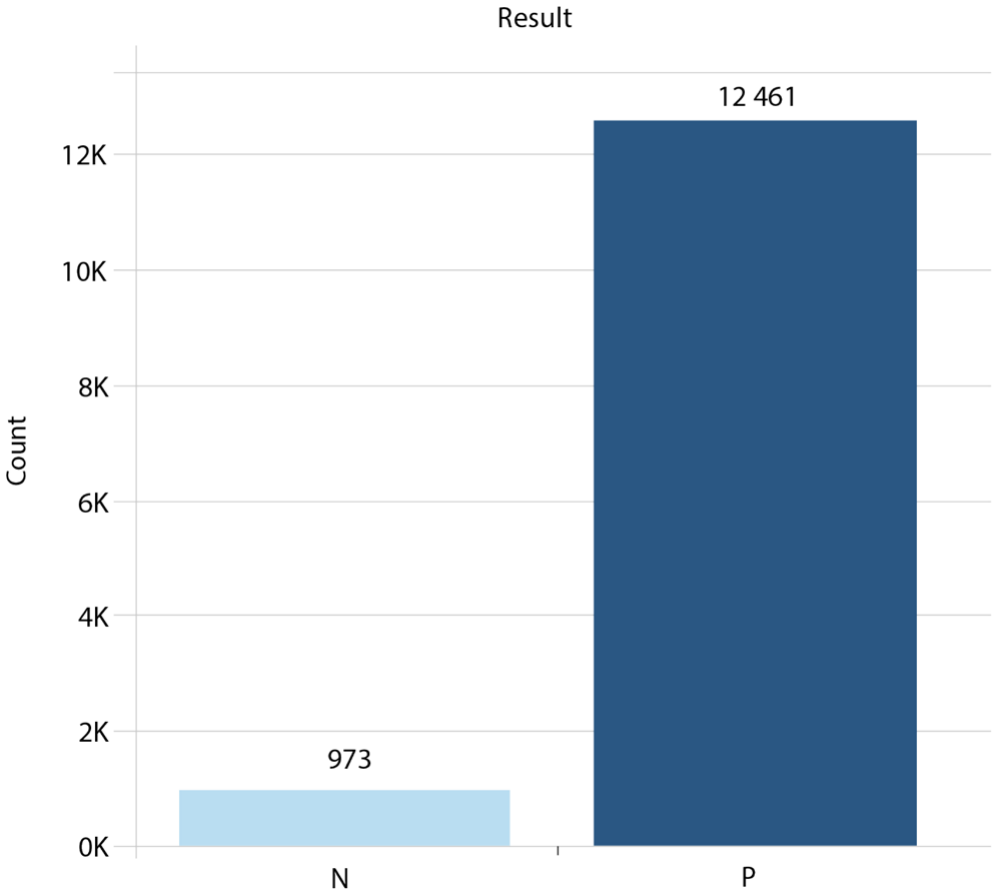
Distribution of patients per test result (target).

Finally, Fig. 6 represents the distribution of patients of this case study in terms of COVID-19 results (the target attribute). This figure shows that the target class distribution is highly imbalanced, with only 7.24% of occurrences yielding a negative result and the remaining 92.76% yielding COVID-19 positive cases.

These numbers reveal that the majority of users who responded to the screening questionnaire provided in the developed web application only wanted to be remotely monitored in case they tested positive for COVID-19. As a result, an unbalanced dataset was produced.

#### Data preparation

Data preparation is the most time-consuming phase of the CRISP-DM process, involving the integration, cleaning, transformation, and sampling of data. The data was initially incorporated and the data cleaning process was applied to identify the existence of duplicate data, missing values, outliers, and inconsistencies.

During the data cleaning process, no duplicate data or outliers were discovered. However, some inconsistencies were identified that had to be addressed. Most inconsistencies were found in the *Temperature* attribute. Because this is a numerical attribute, it is prone to some disparity, such as some patients filling in the values with commas and others rounding them, as well as some patients putting the unit of measurement and others don’t, and in the case of putting the unit, the formatting may differ, i.e. “$^\circ$C”, “degrees”, and “degrees Celsius”. Therefore, all units were removed, and all temperature values were rounded in order to convert them to the Integer type. In addition to this attribute, the attribute *Medication_last_24h* also required a specific transformation process because, since it is a free text field, patients used several designations to refer the same medication. Thus, a lengthy and laborious transformation process was undertaken to ensure that the drug names were consistent.

In addition to data inconsistencies, some missing values were discovered, which were treated by replacing them with the mean value for numeric attributes and the mode value for nominal attributes. Hence, the *Temperature* missing values were replaced by the average value of this attribute. In turn, the missing values of nominal attributes corresponding to the patient’s symptoms were replaced by the most common value, ’No.’

In this stage, the under and over types of sampling methods were evaluated for the definition of different data approaches in order to investigate which type of sampling is better for the classification of COVID-19 cases. In the next stage, Modelling, different scenarios will be generated by selecting certain attributes in order to investigate their impact on the final prediction.

#### Modelling

This phase consisted in the preparation of different DM Models (DMM) using the *RapidMiner* software with the dataset resulting from the Data Preparation stage. As expressed in Eq. (1), each DMM can be described as belonging to an Approach (A), being composed by a Scenario (S), a Missing Values Approach (MVA), a DM Technique (DMT), a Sampling Method (SM), a Data Approach (DA) and a Target (T).

1 $$\begin{aligned} DMM = \{A, S, MVA, DMT, SM, DA, T\} \end{aligned}$$

There was only one target (T), which was the *result* variable. Since Classification was the chosen Approach (A), six different classifiers were selected to be used as DMTs, namely DT, Random Forest (RF), Random Tree (RT), NB, Naïve Bayes-Kernel (NB-K) and Deep Learning (DL). The DL algorithm is a Rapidminer operator implementation that is based on a multi-layer feed-forward artificial neural network trained with stochastic gradient descent using back-propagation on an H2O cluster node.

For each DMT, three Sampling Methods (SM) were tested:

- *Split Validation*, with 80% of the data used for training and the remaining amount for testing.
- *Split Validation*, with 70% of the data used for training and the remaining amount for testing.
- *Cross Validation*, using 10 folds and where all data is used for both training and testing.

Because the class distribution of the target variable was significantly unbalanced, two Data Approaches (DA) were investigated: undersampling and oversampling, with the Synthetic Minority Oversampling Technique (SMOTE) upsampling methodology being used.

In terms of scenarios, the first scenario (S1) includes all attributes. In the second scenario (S2), it was decided to remove the *Thoracalgia* attribute. On the other hand, the third scenario (S3) includes all attributes except the *Shortness_of_smell_taste* attribute, since the loss of smell does not always imply the loss of taste, and vice versa. Therefore, it was decided that it was important to investigate the influence of this attribute on the prediction process. As a result, in this study, the DMMs are defined as follows:

- A = {Classification}
- S = {S1, S2, S3}
- MVA = {N/A, Replace (Average and Replenishment)}
- DMT = {DT, RF, RT, NB, NB-K,DL}
- SM = {Split Validation (80%), Split Validation (70%), Cross Validation (10 folds)}
- DA = {Undersampling, Oversampling (SMOTE upsampling)}
- T = {result}

In total, 216 models were induced according to Eq. (2).

2 $$\begin{aligned} DMM = {1 (A) \times 3 (S) \times 2 (MVA) \times 6 (DMT) \times 3 (SM) \times 2 (DA) \times 1 (T)} \end{aligned}$$

### Evaluation

Since this study focus on a classification approach, the evaluation of each model was based on the confusion matrix, which represents the number of False Positives (FP), False Negatives (FN), True Positives (TP) and True Negatives (TN). From these values, a variety of evaluation metrics can be calculated; however, this study used accuracy (3), precision (4), sensitivity (5) and specificity (6) metrics to support the evaluation and conclusion of the research case. In addition, the Area Under the ROC (Receiver Operating Characteristic) Curve (AUC) was also used to assess the performance of the models. Each of these measures, as well as how they are calculated, is described in detail below.

- **Accuracy:** This indicator calculates the ratio between the instances correctly classified by the predictive model and all classified instances for the correctly TP classified instances, which answers the question:
  *How many patients were accurately classified out of the total?*
  3 $$\begin{aligned} Accuracy = \frac{TP + TN}{TP + TN + FN + FP} \end{aligned}$$
- **Precision:** This parameter measures the proportion of positive occurrences correctly classified by the model to the total number of positive instances, which answers the question:
  *How many of patients who were classified with COVID-19 actually had the disease?*
  4 $$\begin{aligned} Precision = \frac{TP}{TP + FP} \end{aligned}$$
- **Sensitivity:** This metric is considered an integrator indicator and measures the ratio of positive instances correctly classified by the model to the total positive instances, which answers the question:
  *How many COVID-19 patients were successfully predicted out of all of them?*
  5 $$\begin{aligned} Sensitivity = \frac{TP}{TP + FN} \end{aligned}$$
- **Specificity:** Reveals the correctly TN classified instances through the calculation of the proportion of negative instances correctly classified by the model to the total of negative instances, which answers the question:
  *How many healthy patients were correctly predicted?*
  6 $$\begin{aligned} Specificity = \frac{TN}{TN + FP} \end{aligned}$$
- **AUC:** The AUC value is associated with the ROC probabilistic curve and is defined as a measure that informs the model’s ability to distinguish classes, with a higher AUC value indicating that the model predicts 0s as 0s and 1s as 1s more correctly.

## Results

The results analysis is structured according to the different metrics to be evaluated, which were previously described in the evaluation stage of the CRISP-DM cycle. After six predictive algorithms were tested, the best outcome for each metric for each DMT was examined. As mentioned above, three different sampling methods were used to evaluate the models, namely Cross Validation with 10 folds, Split Validation with 70% of data used for training and the remaining for test, and also Split Validation with 80% of data used for training and the remaining for test. Thus, the results of Cross Validation are based on the full dataset and the results of both Split Validation methods are based on the test set.

The best results for each evaluation metric were grouped in different tables and presented by descending order of performance.

Table 2 shows that S1 had the best results in terms of Accuracy, i.e., the assertiveness of patient labeling, when combined with DT, RF, NV-K, and NV techniques, achieving results above 85%. It is worth mentioning that the S3 scenario, the one with the removal of the *Shortness_of_smell_taste* attribute, combined with the DL algorithm also obtained a good result with 89,07% of accuracy. Finally, it is important to note that the best results are not associated with a MVA.

**Table 2:** DMMs with the highest accuracy for each DMT.

DMM	DMT	S	SM	MVA	DA	Accuracy (%)
3	DT	S1	Split Validation (80%)	N/A	SMOTE	96.25
7	RF	S1	Split Validation (70%)	N/A	SMOTE	91.36
105	DL	S3	Split Validation (80%)	N/A	SMOTE	89,07
27	NV-K	S1	Split Validation (80%)	N/A	SMOTE	87.45
19	NV	S1	Split Validation (70%)	N/A	SMOTE	86.32
13	RT	S1	Split Validation (70%)	N/A	SMOTE	68.26

Table 3 shows that excellent results were obtained for the Precision metric, with all models scoring above 90%, indicating that the patients identified with COVID-19 have the condition. The best Precision result was obtained by the DMM3 - {DT, S1, Split Validation (80%),SMOTE} with a result of 99.91%. In contrast, the worst model, DMM83 {RF, S3, Cross Validation, SMOTE}, achieved a precision of 91,97%, which is still quite high.

**Table 3:** DMMs with the highest precision for each DMT.

DMM	DMT	S	SM	MVA	DA	Precision (%)
3	DT	S1	Split Validation (80%)	N/A	SMOTE	99.91
13	RT	S1	Split Validation (70%)	N/A	SMOTE	98.99
21	NV	S1	Split Validation (80%)	N/A	SMOTE	98.80
63	NV-K	S2	Split Validation (80%)	N/A	SMOTE	98.36
103	DL	S3	Split Validation (70%)	N/A	SMOTE	97.57
83	RF	S3	Cross Validation	N/A	SMOTE	91.97

In terms of sensitivity, the predictive models with the best performance were RF, DT, DL, and NV-K, as shown in Table 4. This means that at least 82% of patients infected with SARS-Cov-2 were successfully predicted using these models. In the context of this study, sensitivity is the most important metric for evaluating the models because it is dangerous to predict that a patient infected with SARS-CoV-2 is healthy. It is worth noting that this is the only metric in which DMM3 did not produce the best results, but its performance was still adequate, as it was the second DMM with the highest sensitivity value −92.58%.

**Table 4:** DMMs with the highest sensitivity for each DMT.

DMM	DMT	S	SM	MVA	DA	Sensitivity (%)
9	RF	S1	Split Validation (80%)	N/A	SMOTE	93.42
3	DT	S1	Split Validation (80%)	N/A	SMOTE	92.58
105	DL	S3	Split Validation (80%)	N/A	SMOTE	89.37
28	NV-K	S1	Split Validation (80%)	Replace	SMOTE	82.57
22	NV	S1	Split Validation (80%)	Replace	SMOTE	79.09
18	RT	S1	Cross Validation	Replace	SMOTE	50.80

Specificity, on the other hand, indicates how many healthy patients were correctly predicted. The DMMs with the highest specificity values are listed in Table 5. The DMM3 - {DT, S1, Split Validation (80%), SMOTE}, reveals the best combination to achieve the highest specificity result −99.92%.

**Table 5:** DMMs with the highest specificity for each DMT.

DMM	DMT	S	SM	MVA	DA	Specificity (%)
3	DT	S1	Split Validation (80%)	N/A	SMOTE	99.92
13	RT	S1	Split Validation (70%)	N/A	SMOTE	99.63
21	NV	S1	Split Validation (80%)	N/A	SMOTE	99.12
63	NV-K	S2	Split Validation (80%)	N/A	SMOTE	98.76
103	DL	S3	Split Validation (70%)	N/A	SMOTE	98.18
83	RF	S3	Cross Validation	N/A	SMOTE	92.35

Finally, the DMMs with the best AUC values are listed in Table 6. The highest AUC value was obtained with DMM7 - {RF, S1, Split Validation (70%), SMOTE}. On the contrary, DMM51 - {RT, S2, Split Validation (80%), SMOTE} performed poorly in terms of AUC, achieving a value closer to a random classifier, which yields an AUC of 0.5.

**Table 6:** DMMs with the highest AUC for each DMT.

DMM	DMT	S	SM	MVA	DA	AUC
7	RF	S1	Split Validation (70%)	N/A	SMOTE	0.972
3	DT	S1	Split Validation (80%)	N/A	SMOTE	0.963
105	DL	S3	Split Validation (80%)	N/A	SMOTE	0.958
19	NV	S1	Split Validation (70%)	N/A	SMOTE	0.951
25	NV-K	S1	Split Validation (70%)	N/A	SMOTE	0.950
51	RT	S2	Split Validation (80%)	N/A	SMOTE	0.687

## Discussion

According to the majority of the results, Split Validation was the most successful SM in this study. Split validation is useful when dealing with large datasets and complex preparation processes because it allows for some uncertainty about the model’s robustness to be accepted. Cross Validation is more computationally complex when the data set is large, which results in a slower overall computational performance.

In terms of scenarios, it can be observed that scenario S1, which included all of the attributes, produced the best results, followed by scenario S3, which made it into seven of the best performing models and scenario S2, which made it into three of the best performing models. In other words, when assessing the diagnosis of COVID-19 cases, all of the attributes used in this study should be considered. In what concerns the MVA, as shown in the example above, it can be seen that models perform better in general when missing values are not replaced by the mean or mode value, depending on whether the missing values are numerical or nominal respectively. This is not surprising given the large number of missing values in the dataset, the majority of which are categorical attributes corresponding to the progression of symptoms that are extremely subjective in nature.

By far, the best results were obtained when using the Oversampling approach, namely the SMOTE Upsampling technique. There was some experimentation with the Undersampling method, but it did not produce the best results. This was most likely due to the minority class being extremely small in comparison to the majority class, resulting in a significant loss of critical data. Regarding algorithms, DT was the one that achieved the best results. The performance of the remaining methods cannot be easily compared because of the problem’s complexity.

**Figure 7:**
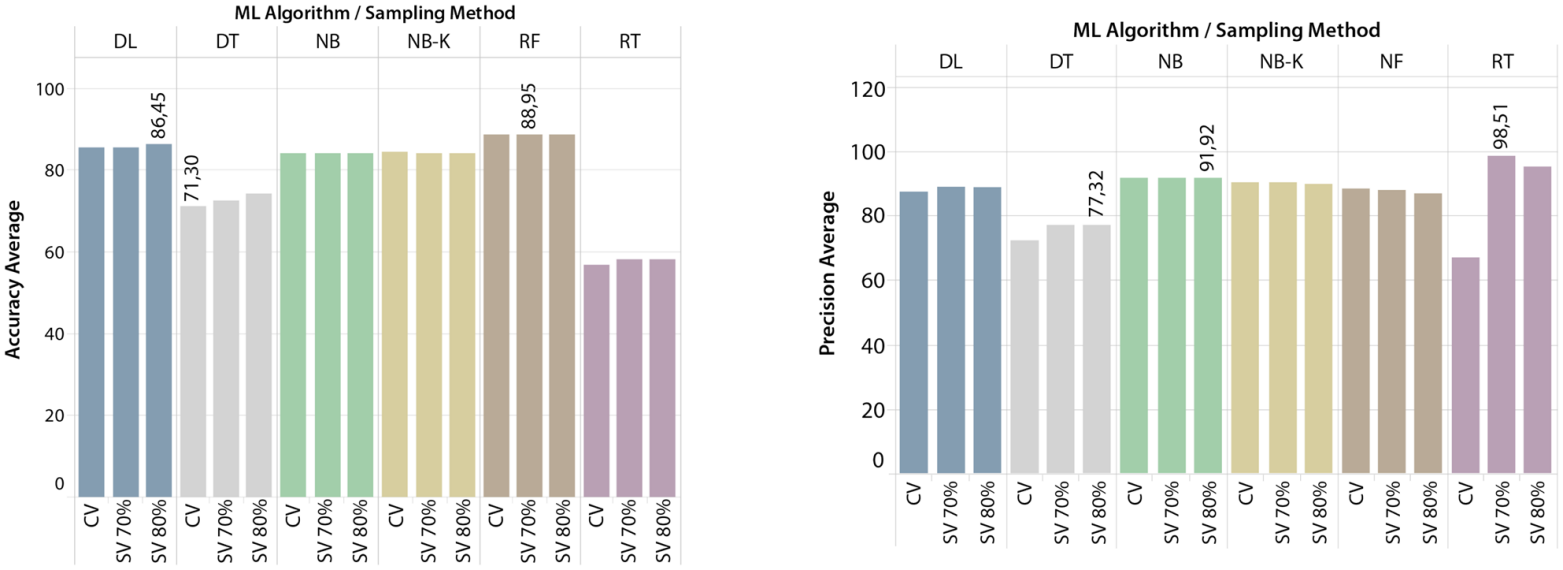
Average accuracy and precision of 216 tested models per DMT and SM.

**Figure 8:**
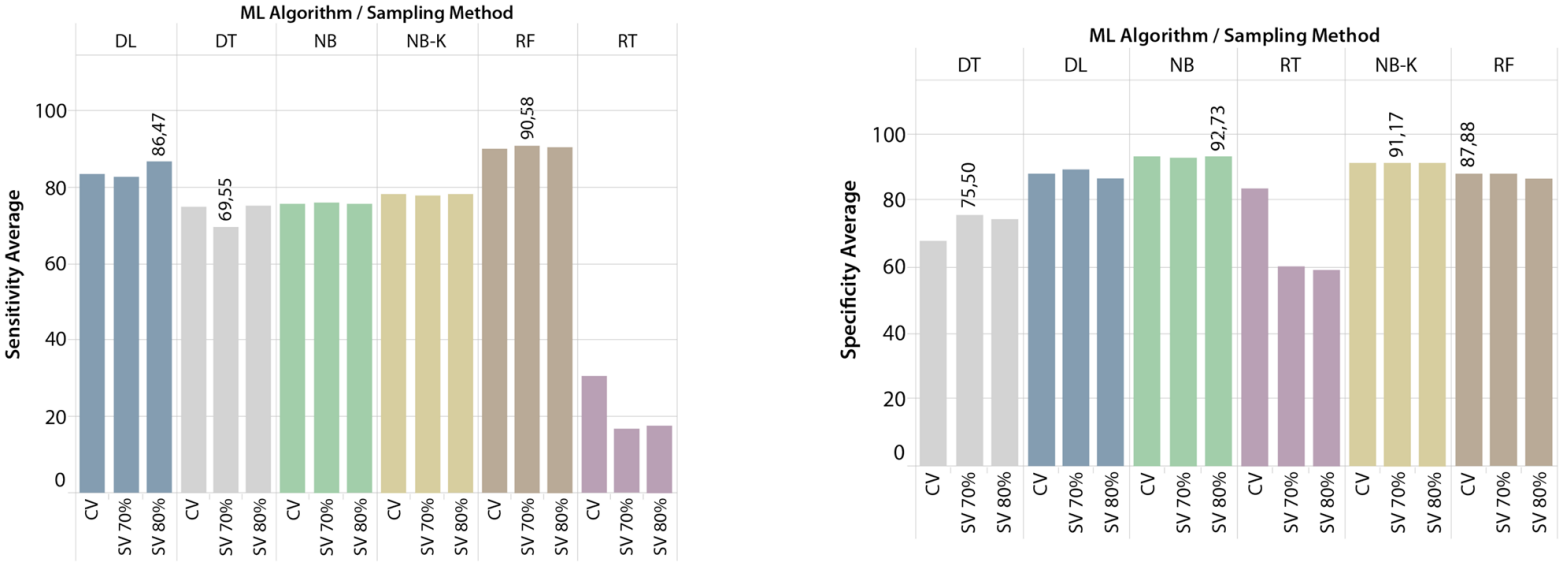
Average Sensitivity and Specificity of 216 tested models per DMT and SM.

Dashboards for each indicator were created using the average of all models trained by each forecasting algorithm and sampling strategy, making it easier to evaluate the results in general. The dashboards are presented in Figs. 7 and 8.

As shown in Fig. 7, RF with Split Validation (70%) and Deep Learning with Split Validation (80%) were the two DMMs that obtained the highest average Accuracy. In terms of Precision, the DMM using the RT algorithm and the Split Validation (70%) method achieved the highest result (98.51%), followed by the DMM using the NB classifier and the Split Validation (80%) method (91.92%).

Similarly to the Accuracy metric, Fig. 8 shows that Deep Learning (86.47%) and Random Forest (90.58%) stand out among the other tested algorithms in terms of Sensitivity. On the other hand, it is important to emphasise the RT algorithm’s low results, all below 50%. Regarding the Specificity metric, as shown in Fig. 8, the NB-K and NB algorithms produced the best overall results, with values ranging between 91 and 93% of all possible outcomes. The RT algorithm, on the other hand, produced extremely low averages. As mentioned earlier, when the sampling method Split validation is used instead of Cross validation, the average of the indicators produces better results.

Hence, the findings of this study have proved that the use of openEHR is relevant and necessary to ensure the value of clinical data, which is emphasised by the current pandemic context, in which it is emerging to extract new knowledge in a short period of time. This study also demonstrates that it is possible to use ML techniques to accurately extract clinical insights, always taking into account the requirement to obtain high sensitivity values, safeguarding patients’ health as much as possible. Furthermore, this research shows that the use of clinical terminologies, specifically openEHR, created the optimal foundation for an efficient knowledge extraction, generating interpretable data and reducing its heterogeneity and dispersion, thus demonstrating that in order to generate useful knowledge and draw relevant and valuable insights, we must first focus on the quality of the raw material, that is, on the data. Thus, we believe that clinical data structuring and standardisation are critical aspects in improving the efficiency of medical practise and differentiating factors in the quality of the services provided. In the future, we believe that openEHR will be essential to any health system.

## Conclusions

IT systems are transforming the healthcare industry in ways never thought before, from the discovery of cures for diseases and the development of new treatment techniques to the improvement of patients’ diagnosis and the follow-up of clinical situations. As a result, the benefits of using IT approaches in clinical settings, such as improving patients’ quality of care and optimising health institutions’ resources, have become widely recognised, from health centres to large-scale hospitals around the world.

With the COVID-19 pandemic’s exponential growth and development, the health system of each country was compelled to implement numerous changes capable of responding quickly and efficiently to the new cases that appeared on a daily basis. By the time the first cases of this pandemic were discovered in Portugal, a pilot project was already being conducted at CHUP on the use of globally recognised clinical standards, particularly openEHR. Given the numerous benefits that these standard specifications bring to the structuring of clinical data, including data integrity and the reduction of data loss and/or misinterpretation, it would be naive not to take advantage of these open specifications and clinical models in the new pandemic context. Thus, screening questionnaires based on openEHR structures were included in this Portuguese hospital prior to the provision of a health service or a COVID-19 test, with the goal of monitoring patients suspected or infected with SARS-CoV-2. The goal of this research is to use various machine learning algorithms to extract useful information from the data collected in these questionnaires.

Accordingly, one of the most promising outcomes of this project is the discovery of how quickly and efficiently globally recognised methodologies and standards such as openEHR can be implemented as well as how they can interoperate with LSs already in place at each health institution. Another meaningful finding of this study concerns the quality of clinical information obtained through the use of openEHR in the service of medicine, so as to improve the quality of healthcare delivery, as well as the value that these data can provide when used to support clinical decision-making.

Through the methodologies and investigation strategies chosen, it was possible to define a valid strategy starting from topics and key ideas that became more solid and justified with the revision of the literature. Additionally, this study demonstrated that the data generated by this new system can be used to train predictive ML models with sound performance. In this context, the openEHR standard was quickly adapted and implemented in a COVID-19 patient circuit, and the data from inquired patients was used to feed forecasting models based on the patients’ symptoms and current health status.

In terms of results, practically all models achieved accuracy rates over 80%, which is remarkably impressive. DMM number 3 had the best results with 96.25% accuracy, 99.91% precision, 92.58% sensitivity, 99.92% specificity, and 0.963 AUC, combining the dataset with all symptoms, the Split Validation (80%) method, and the Decision Tree algorithm without replacing the missing values. Hence, the predictive model could be implemented in a CDSS to assist healthcare professionals after collecting more data and subjecting the models to additional testing and more rigorous evaluations.

In the future, some occurrences in the items of the openEHR templates developed could be changed to obligate users to fill out the corresponding form fields, thus reducing the number of missing values in the dataset and improving the trustworthiness of the produced results. Furthermore, it would be important to reduce the free text fields, which is easy to accomplish using specific datatypes provided by openEHR. In this case, for example, the *Medication_last_24h* should be a *DV_CODED_TEXT* datatype to narrow the users’ responses to the options provided in the form. As a result, the data preparation stage would be less time-consuming and difficult, and the results would be more accurate. In addition, in the future, it will be interesting to validate the findings of this research with a validation set that is completely independent of the training and test data, in order to verify the adequacy of the developed models, for a better and more complete evaluation of the behavior of the models. Finally, while the results are promising, the amount of data used in this experiment is insufficient to draw firm conclusions. Data from different healthcare institutions should be collected in the future, not only to create a richer and more varied dataset, but also to achieve a more balanced distribution of classes, avoiding the need for data sampling techniques and making the models more reliable and realistic. Furthermore, one limitation of this study is that the data acquisition stage does not consider the collection window. For example, a patient who reports symptoms on day 1 but tests positive on day 14 is in a very different situation than a patient who reports symptoms several days after testing positive. Hence, the data collection window should be considered in future work to improve the accuracy and make it reproducible for similar settings.

## Data Availability

The dataset analysed during the current study is not publicly available due to the Administrative Council of Centro Hospitalar Universitário do Porto’s authorization for research purposes only and not for publication, but are available from the corresponding author on reasonable request.

## Code availability

The computer code associated with the algorithms used to generate the results of this manuscript is available from the corresponding author upon request.
